# 1 V Tunable High-Quality Universal Filter Using Multiple-Input Operational Transconductance Amplifiers

**DOI:** 10.3390/s24103013

**Published:** 2024-05-09

**Authors:** Montree Kumngern, Fabian Khateb, Tomasz Kulej, Boonying Knobnob

**Affiliations:** 1 Department of Telecommunications Engineering, School of Engineering, King Mongkut’s Institute of Technology Ladkrabang, Bangkok 10520, Thailand; montree.ku@kmitl.ac.th; 2 Department of Microelectronics, Brno University of Technology, Technická 10, 601 90 Brno, Czech Republic; 3 Faculty of Biomedical Engineering, Czech Technical University in Prague, nám. Sítná 3105, 272 01 Kladno, Czech Republic; 4 Department of Electrical Engineering, Brno University of Defence, Kounicova 65, 662 10 Brno, Czech Republic; 5 Department of Electrical Engineering, Czestochowa University of Technology, 42-201 Czestochowa, Poland; kulej@el.pcz.czest.pl; 6 Faculty of Engineering, Rajamangala University of Technology Thanyaburi, Pathum Thani 12110, Thailand; kboonying@rmutt.ac.th

**Keywords:** universal filter, voltage-mode circuit, operational transconductance amplifier

## Abstract

This paper presents a new multiple-input single-output voltage-mode universal filter employing four multiple-input operational transconductance amplifiers (MI-OTAs) and three grounded capacitors suitable for low-voltage low-frequency applications. The quality factor (*Q*) of the filter functions can be tuned by both the capacitance ratio and the transconductance ratio. The multiple inputs of the OTA are realized using the bulk-driven multiple-input MOS transistor technique. The MI-OTA-based filter can also offer many filtering functions without additional circuitry requirements, such as an inverting amplifier to generate an inverted input signal. The proposed filter can simultaneously realize low-pass, high-pass, band-pass, band-stop, and all-pass responses, covering both non-inverting and inverting transfer functions in a single topology. The natural frequency and the quality factors of all the filtering functions can be controlled independently. The natural frequency can also be electronically controlled by tuning the transconductances of the OTAs. The proposed filter uses a 1 V supply voltage, consumes 120 μW of power for a 5 μA setting current, offers 40 dB of dynamic range and has a third intermodulation distortion of −43.6 dB. The performances of the proposed circuit were simulated using a 0.18 μm TSMC CMOS process in the Cadence Virtuoso System Design Platform to confirm the performance of the topology.

## 1. Introduction

An operational transconductance amplifier (OTA) is a voltage-controlled current source that offers numerous advantages in circuit design, such as providing electronic tuning capability, easy implementation of the OTA structure, and the powerful ability to realize various applications. In addition, OTA-based circuits are usually absent from resistor requirements, making them suitable for integrated circuit (IC) implementation [1,2].

Biquad filters are very useful blocks for applications in measurement, communication, and control systems. From the general form of second-order filter functions [3], there are five frequency responses that are possible to obtain, namely low-pass filter (LPF), high-pass filter (HPF), band-pass filter (BPF), band-stop filter (BSF), and all-pass filter (APF). These are the so-called five standard filtering functions. A biquad filter can be used to realize high-order filters by cascading multiple first-order and second-order sections, as used in phase-lock loops (PLL) for loop filtering (usually an LP filter), FM stereo demodulators (usually LP and BP filters), and crossover networks in three-way high fidelity (usually LP, BP, and HP filters) [3]. A filter that can provide several second-order filters in a single topology is classified as a universal filter. There are many universal filters available in the literature using varying active devices, such as second-generation current conveyors (CCIIs) [4,5,6] and current feedback operational amplifiers (CFOAs) [7,8,9]. Unfortunately, these filters lack electronic tuning capabilities, which is important when parameters such as the natural frequency and quality factor deviate by process–voltage–temperature (PVT) variations. Some universal filters that offer the possibility of electronic tuning and minimal active elements have been introduced by using the voltage differencing inverting buffered amplifier (VDIBA) [10,11] and inverters [12]. However, these filters supply the input signals through capacitors, and therefore, an additional buffer circuit is required, and these capacitors become floating.

This work is focused on a universal filter with electronic tuning capability that utilizes an operational transconductance amplifier (OTA) as the active element. There are many universal filters using OTAs as active elements available in the literature; for example, see [13,14,15,16,17,18,19,20,21,22,23,24,25,26,27,28,29,30,31,32,33,34,35,36,37,38,39,40,41,42,43,44,45,46,47,48,49]. The circuits in [13,14,15,16] are current-mode filters, the circuits in [17,18,19,20,21,22,23,24,25,26,27,28,29,30,31,32,33,34,35] are voltage-mode filters, and the circuits in [35,36,37,38,39,40,41,42] are mixed-mode filters. Considering the input and output terminals, the circuits in [17,18,19,20,21,22,39] are single-input multiple-output (SIMO) filters, the circuits in [15,16,23,24,25,26,27,28,29,40,41] are multiple-input single-output (MISO) filters, and the circuits in [13,14,30,31,32,33,34,35,36,37,38,42] are multiple-input multiple-output (MIMO) filters. Compared with SIMO filters, MISO and MIMO filters usually employ fewer active devices because the variant filtering functions of these filters can be obtained by appropriately selecting the input and/or output terminals. This work is focused on utilizing the MISO filter so that parameters such as the natural frequency and the quality factor can be electronically and independently controlled. Considering the MIMO and MISO filters in [13,14,15,16,23,24,25,26,27,28,29,30,31,32,33,34,35,36,37,38,40,41,42], these filters suffer from some drawbacks:

(i) They require additional circuits at the input, such as a SIMO current follower circuit [13,14,15,16].
(ii) They use a floating capacitor or floating resistor [23,28,31,32].
(iii) They do not provide the non-inverting and inverting transfer functions of LP, HP, BP, BS, and AP filters [23,24,25,26,27,28,29,30,31,32,33,34,35,36,37,38,40,41].
(iv) They do not provide independent tunable control of the natural frequency and the quality factor [23,24,25,27,29,30,31,32,33,38,40,41].
(v) They require inverted input signals to obtain some transfer functions [24,31,37,38].

It should be noted that these universal filters are not designed for low-voltage low-power signal-processing applications. Nowadays, low-voltage low-power filters are required for biomedical applications, such as biosensors [3]. Universal filters using OTAs operating with a low supply voltage and with low power consumption are available in the literature [42,43,44,45,46,47]. However, these configurations cannot benefit from independent and electronic control of the quality factor and the natural frequency and cannot provide a high-quality (high-*Q*) filter. In modern applications, high-*Q* filters are strictly desirable for processing weak signals, such as the detection, measurement, and quantification of biomedical signals [48,49]. The bio-signal has the attributes of a low amplitude and a low frequency (≤10 kHz).

This paper presents a low-voltage low-power universal biquadratic filter that allows the natural frequency and quality factor to be independently and electronically controlled. A high-*Q* filter can also be obtained. The filter is realized using multiple-input operational transconductance amplifiers (MI-OTAs). In the filter’s input differential stage, the MI-OTAs are realized using the multiple-input MOS transistor technique (MI-MOST), which obtains a minimal differential pair and minimal power consumption. Using an MI-OTA-based filter shows that both the non-inverting and the inverting transfer functions of LP, HP, BP, BS, and AP filters can be obtained without inverted input signals. The proposed filter uses a 1 V supply voltage and 120 μW of power consumption for a 5 μA setting current. The filter was designed and simulated in the Cadence Virtuoso environment using 0.18 μm TSMC CMOS technology.

## 2. Circuit Description

### 2.1. Multiple-Input OTA

**Figure 1:**
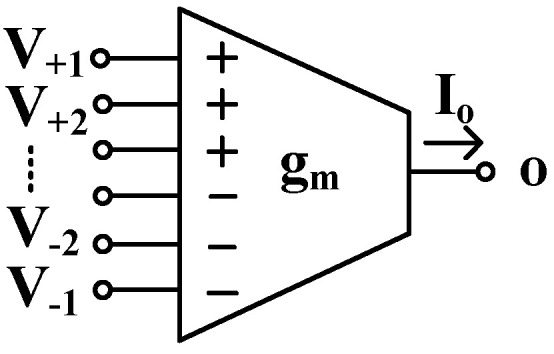
Electrical symbol of the MI-OTA.

The multiple-input OTA is used to realize the filter application. Its circuit symbol is shown in Figure 1. Ideally, the transfer characteristic of this OTA is given by the following equation:

(1) $$I_{o}=g_{m}\left(V_{+1}+V_{+2}+\ldots +V_{+n}-V_{-1}-V_{-2}-\ldots -V_{-n}\right)$$

where $I_{o}$ is the output current, and $g_{m}$ is the small-signal transconductance. Note that the circuit in the general case has *n* non-inverting and *n* inverting inputs; thus, its input voltage can be considered as the difference of two sums of voltages applied to the non-inverting $V_{+1,\ldots ,+n}$ and inverting $V_{-1,\ldots ,-n}$ inputs, respectively.

**Figure 2:**
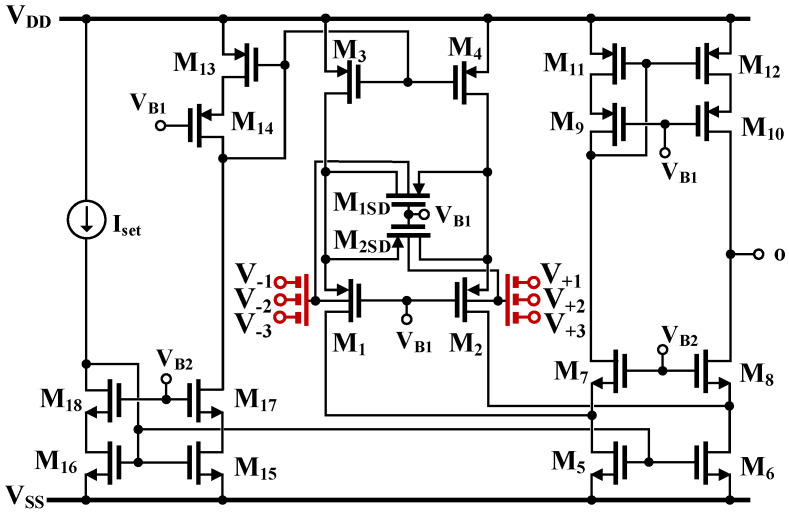
CMOS realization of the MI-OTA using the MIBD-MOST technique.

**Figure 3:**
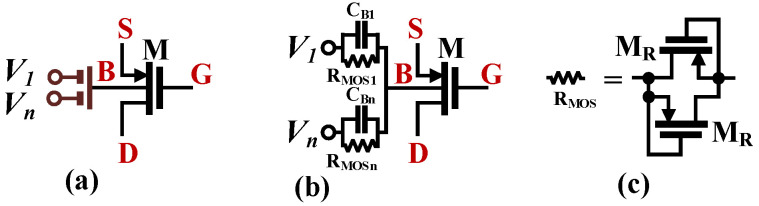
The MIBD-MOST technique: (**a**) symbol, (**b**) realization, and (**c**) R_MOS_ realization.

The transistor-level schematic of the OTA proposed in this work with *n* = 3 is shown in Figure 2. The circuit consists of an OTA with folded-cascode topology with a linearized input stage consisting of the transistors M_1_–M_2_ and M_1SD_, M_2SD_, and biased by the current sinks M_3_ and M_4_. The transistors M_13_–M_18_ were used for biasing. The multiple inputs were realized in a simple way by adding a capacitive voltage divider to the transistors M_1_ and M_2_, thus creating a multiple-input device, as shown in Figure 3. The input capacitors C_Bi_ were bypassed by large R_MOSi_ resistances, which were realized as an anti-parallel connection of MOS transistors operating in a cutoff region. The large resistances provided the DC biasing of the M_1_ and M_2_ gates.

The linearization technique used in this work is similar to the technique with gate-driven input stages operating in strong inversion introduced in [50]. However, in this work, the bulk-driven devices operating in weak inversion were applied to the proposed structure. Operation in weak inversion generally leads to a narrower linear range compared to the strong inversion version of the circuit. On the contrary, the use of bulk-driven terminals extends the linear range compared to the gate-driven realization. Moreover, the input capacitive divider further extends the linear range. The result is the relatively large linear range of the OTA, even for weakly inverted devices biased with very low currents. A similar input stage was first described and verified experimentally in [51,52]. The M_1SD_ and M_2SD_ transistors operate in a triode region, introducing negative feedback to the input pair M_1_ and M_2_. Controlling their characteristics by the signals seen at the gates of the main transistors of the pair M_1_ and M_2_ further improves the linearity of the input stage [51].

Assuming that all the capacitances C_Bi_ are identical, the small-signal transconductance of the OTA is given by:

(2) $$g_{m}=\frac{\eta }{n}\cdot \frac{4k}{4k+1}\cdot \frac{I_{set}}{n_{p}U_{T}}$$

where $\eta =g_{mb1,2}/g_{m1,2}$ is the bulk to gate transconductance ratio at the operating point, n is the number of input terminals, n_p_ is the subthreshold slope factor for the p-channel transistors, *U*_T_ is the thermal potential, I_set_ is the biasing current and *k* is the ratio of aspect ratios of the triode-region transistors M_1SD_, M_2SD_ and the main transistors of the input pair M_1_ and M_2_:

(3) $$k=\frac{{\left(W/L\right)}_{1SD,2SD}}{{\left(W/L\right)}_{1,2}}$$

Note that the best linearity performance is obtained for *k* = 0.5 [51]. In such a case, the circuit transconductance can be expressed as:

(4) $$g_{m}=\frac{2}{3}\cdot \frac{\eta }{n}\cdot \frac{I_{set}}{n_{p}U_{T}}$$

The use of an input capacitive divider and bulk-driven devices extends the linear range of the OTA, but on the other hand, it increases the input noise and decreases the voltage gain. For instance, with η=1/3 and 3 inputs, the circuit transconductance is lowered 9 times as compared to a gate-driven input pair. Consequently, the low-frequency voltage gain of the circuit is decreased by around 19 dB. To counteract this effect, we applied a cascode high-impedance output stage, composed of the transistors M_5_–M_12_. With the applied output stage, the low-frequency gain of the OTA can be approximated as:

(5) $$A_{V}≅g_{m}[\left(g_{m8}r_{ds8}r_{ds6}\right)||\left(g_{m10}r_{ds10}r_{ds12}\right)]$$

Consequently, this gain is improved by the factor of $g_{m}r_{ds}$ (intrinsic voltage gain of an MOS transistor), which for the applied technology and operating point exceeds 30 dB.

As was already mentioned, the applied technique increases the linear range of the OTA. On the other hand, however, it increases its input noise due to signal attenuation. Since the input noise is increased in the same proportion, the dynamic range (DR) of the OTA remains unchanged and is equal to the DR of the GD OTA with a single differential input and the applied linearization technique. Nevertheless, the larger linear range allows for avoiding hard nonlinearities for the large voltage swings and V_DD_, as applied in the considered design.

### 2.2. Proposed Tunable High-Q Voltage-Mode Universal Filter

**Figure 4:**
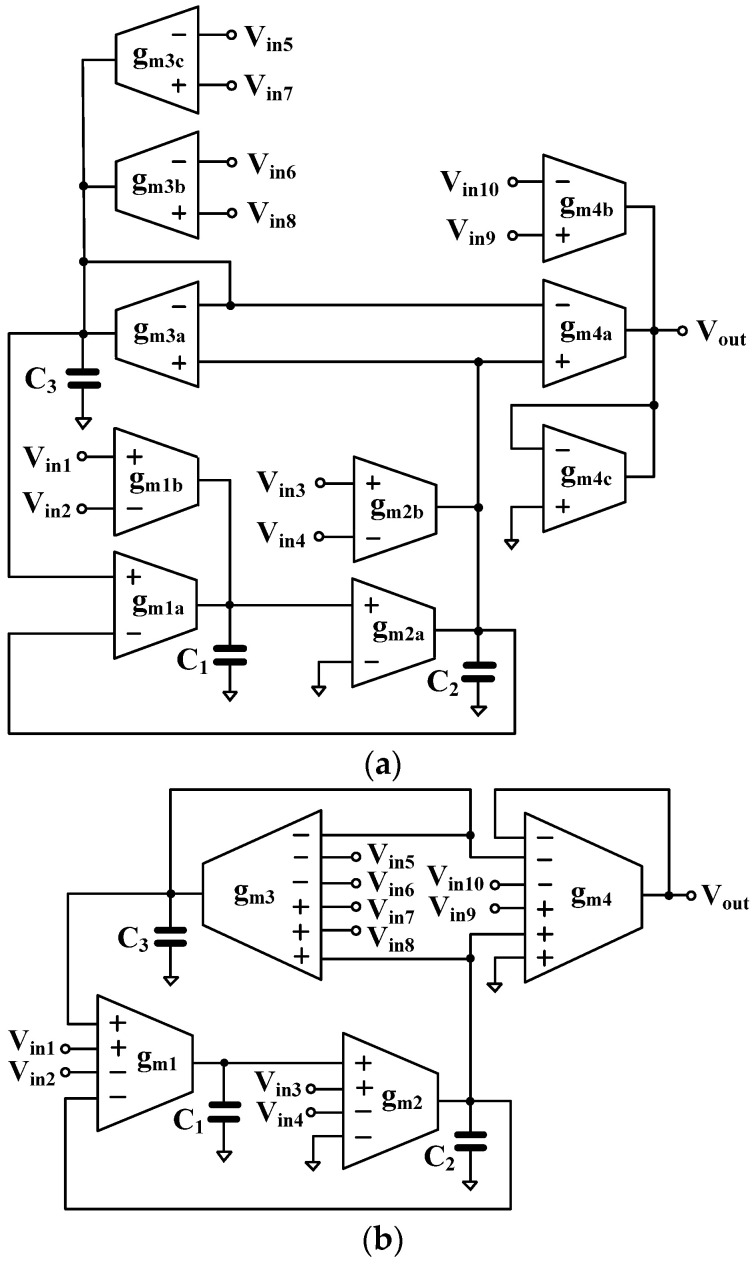
Proposed tunable high-*Q* voltage-mode universal filter using (**a**) conventional OTAs and (**b**) MI-OTAs.

Figure 4 shows the proposed tunable high-*Q* voltage-mode universal filter using OTAs. Figure 4a shows the proposed voltage-mode universal filter using conventional OTAs and Figure 4b shows the proposed voltage-mode universal filter using MI-OTAs. It should be noted from Figure 4a,b that the universal filter using MI-OTAs has a significantly reduced number of OTAs (10 OTAs vs. 4 MI-OTAs). The input terminals of the universal filter in Figure 4b are connected to the high-input impedance of the OTA; thus, the proposed universal filter offers high input impedance, which is ideal for voltage-mode circuits. The output impedance can be given by ${1/g}_{m4}$.

Letting $g_{m1a}=g_{m1b}=g_{m1}$, $g_{m2a}=g_{m2b}=g_{m2}$, $g_{m3a}=g_{m3b}=g_{m3c}=g_{m3}$, $g_{m4a}=g_{m4b}=g_{m4c}=g_{m4}$, and using nodal analysis, the output voltage of Figure 4a,b can be given by:

(6) $$V_{out}=\frac{\left\{\begin{array}{c} g_{m1}g_{m2}\left(V_{in1}-V_{in2}\right)+sC_{1}g_{m2}\left(V_{in3}-V_{in4}\right)\\ +s\frac{C_{1}C_{2}g_{m3}}{C_{3}}\left(V_{in5}+V_{in6}-V_{in7}-V_{in8}\right)\\ +D\left(s\right)\left(V_{in9}-V_{in10}\right) \end{array}\right\}}{D\left(s\right)}$$

where $D\left(s\right)=s^{2}C_{1}C_{2}+s\frac{C_{1}C_{2}g_{m3}}{C_{3}}+g_{m1}g_{m2}$.

The variant filtering functions are shown in Table 1. It should be noted that the variant non-inverting and inverting transfer functions of the LPF, BPF, HPF, BSF, and APF can be obtained without inverted input signal requirements. For the BPF, if the input signals are *V_in_*_3_ and *V_in_*_4_, varying the quality factor will increase the gain of the transfer functions. Conversely, if the input signals are *V_in_*_5_ or *V_in_*_6_ and *V_in_*_7_ or *V_in_*_8_, varying the quality factor will not affect the gain of the transfer functions.

Letting *V_in_*_7_ = *V_in_*_8_ = *V_in_*_9_ = *V_in_*, *V_out_* = *V_AP_*_+_, the transfer function of the non-inverting APF can be expressed as in (7), and letting *V_in_*_5_ = *V_in_*_6_ = *V_in_*_10_ = *V_in_*, *V_out_* = *V_AP_*_−_, the transfer function of the inverting APF can be expressed as in (8).

(7) $$\frac{V_{AP+}}{V_{in}}=\frac{s^{2}C_{1}C_{2}-s\frac{C_{1}C_{2}g_{m3}}{C_{3}}+g_{m1}g_{m2}}{s^{2}C_{1}C_{2}+s\frac{C_{1}C_{2}g_{m3}}{C_{3}}+g_{m1}g_{m2}}$$

(8) $$\frac{V_{AP-}}{V_{in}}=\frac{-s^{2}C_{1}C_{2}+s\frac{C_{1}C_{2}g_{m3}}{C_{3}}-g_{m1}g_{m2}}{s^{2}C_{1}C_{2}+s\frac{C_{1}C_{2}g_{m3}}{C_{3}}+g_{m1}g_{m2}}$$

These transfer functions can be used to express the magnitudes and phase responses of APFs.

The natural frequency ($\omega_{o}$) and the quality factor (*Q*) can be given by:

(9) $$\omega_{o}=\sqrt{\frac{g_{m1}g_{m2}}{C_{1}C_{2}}}$$

(10) $$Q=\frac{C_{3}}{g_{m3}}\sqrt{\frac{g_{m1}g_{m2}}{C_{1}C_{2}}}$$

The parameter $\omega_{o}$ can be controlled electronically by $g_{m1}$ and $g_{m2}$ and the parameter *Q* can be controlled by $C_{3}$ and/or $g_{m3}$. If $C_{3}$ is used as a parameter, $C_{1}$ and $C_{2}$ could be constant ($C_{1}$ = $C_{2}$), and if $g_{m3}$ is used as a parameter, $g_{m1}$ and $g_{m2}$ could be constant ($g_{m1}=g_{m2}$). Thus, the parameter *Q* can be tuned by varying the values of the capacitance and resistance.

### 2.3. Effects of the Nonidealities of the MI-OTA

**Figure 5:**
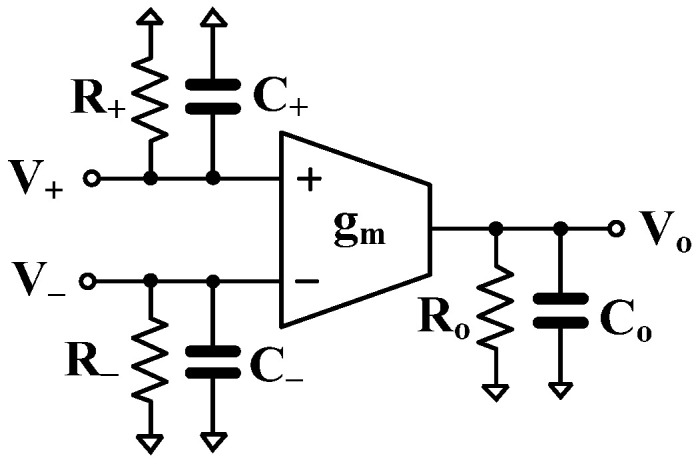
Nonideal structure of the OTA.

Figure 5 shows the nonideal model of the OTA [53]. There are three components that have been considered: (i) the input capacitances *C*_+_, *C*_−_, and input resistances *R*_+_, *R*_−_; (ii) the output capacitance *C_o_* and output resistance *R_o_* (or conductance $g_{o}$); and (iii) the frequency-dependent transconductance $g_{m}$.

The frequency-dependence of $g_{m}$ ($g_{mn}$) can be approximated [54] as:

(11) $$g_{mn}=g_{m}\left(1-s\tau \right)$$

where $\tau =1/\omega_{p}$ and $\omega_{p}$ denotes the second pole of the OTA.

The first consideration can be rewritten by using (9) and the denominator of (6) as:

(12) $$s^{2}C_{1}C_{2}\left(1-\frac{g_{m3}\tau_{3}}{C_{3}}+\frac{{g_{m1}g_{m2}\tau }_{1}\tau_{2}}{C_{1}C_{2}}\right)\;+s\frac{C_{1}C_{2}g_{m3}}{C_{3}}\left(1-\frac{C_{3}g_{m1}g_{m2}}{C_{1}C_{2}g_{m3}}\left(\tau_{1}-\tau_{2}\right)\right)+g_{m1}g_{m2}$$

It can be seen that the parasitic poles ($\tau_{i}$) of the *i*-th OTA affect the filter performance. The influence of the parasitic pole can be neglected if the following conditions are met:

(13) $$\left.\begin{array}{c} \frac{g_{m3}\tau_{3}}{C_{3}}+\frac{{g_{m1}g_{m2}\tau }_{1}\tau_{2}}{C_{1}C_{2}}\ll 1\\ \frac{C_{3}g_{m1}g_{m2}}{C_{1}C_{2}g_{m3}}\left(\tau_{1}-\tau_{2}\right)\ll 1 \end{array}\right\}$$

Next, the parasitic capacitances and resistances (or conductance) have been considered by letting the transconductance $g_{m}$ be ideal. Considering Figure 4b, the values of the capacitors C_1_, C_2_, and C_3_ can be represented, respectively, by $C_{1}^{\prime }$, $C_{2}^{\prime }$, and $C_{3}^{\prime }$, where $C_{1}^{\prime }=C_{1}+C_{o1}+C_{+2}$, $C_{2}^{\prime }=C_{2}+C_{o2}+C_{-1}+C_{+3}+C_{+4}$, and $C_{3}^{\prime }=C_{3}+C_{o3}+C_{+1}+C_{-3}+C_{-4}$, where $C_{oj}$ is the output capacitance of the *j*-th $g_{m}$, and $C_{+j}$ and $C_{-j}$ are the input capacitances of the *j*-th $g_{m}$ (*j* = 1, 2, 3, 4).

When the parasitic resistances are considered, the capacitors $C_{1}^{\prime }$, $C_{2}^{\prime }$, and $C_{3}^{\prime }$ are expressed, respectively, by $C_{1}^{\prime \prime }$, $C_{2}^{\prime \prime }$, and $C_{3}^{\prime \prime }$, where $C_{1}^{\prime \prime }=C_{1}^{\prime }//R_{o1}//R_{+2}$, $C_{2}^{\prime \prime }=C_{2}^{\prime }//R_{o2}//R_{-1}//R_{+3}//R_{+4}$, $C_{3}^{\prime \prime }=C_{3}^{\prime }//R_{o3}//R_{+1}//R_{-3}//R_{-4}$, where *R_oj_* is the output resistance of the j-th $g_{m}$, R_+j_ and R_−j_ are the input resistances of the j-th $g_{m}$ (j = 1, 2, 3, 4).

The parasitic effects on the natural frequency and the quality factor of the proposed universal filter can be avoided by choosing:

(14) $$\left.\begin{array}{c} C_{1}\gg \left(C_{o1}+C_{+2}\right)\;\;\;\;\;\;\;\;\;\;\;\;\;\;\;\;\;\;\;\;\;\;\;\\ C_{2}\gg \left(C_{o2}+C_{-1}+C_{+3}+C_{+4}\right)\\ C_{3}\gg \left(C_{o3}+C_{+1}+C_{-3}+C_{-4}\right) \end{array}\right\}$$

## 3. Simulation Results

The proposed MI-OTA and the filter application were simulated in the Cadence Virtuoso System Design Platform using the 0.18 μm CMOS technology from TSMC (Taiwan Semiconductor Manufacturing Company, Taiwan). The aspect ratio of the MOS transistors of the MI-OTA in Figure 1 is listed in Table 2. The voltage supply was 1 V (V_DD_ = −V_SS_ = 0.5 V). The proposed MI-OTA consumed 30 μW for a 5 μA setting current.

**Figure 6:**
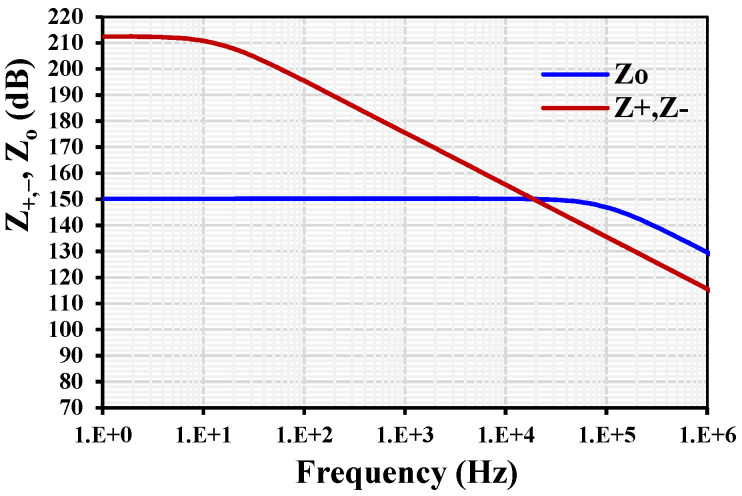
The parasitic impedances of the MI-OTA.

The parasitic impedances of the MI-OTA are shown in Figure 6, where *R*_+,−_ = 42 GΩ, *C*_+,−_ = 0.25 pF for the input terminal, and *R*_o_ = 32.4 MΩ, *C*_o_ = 52.8 fF for the output terminal.

**Figure 7:**
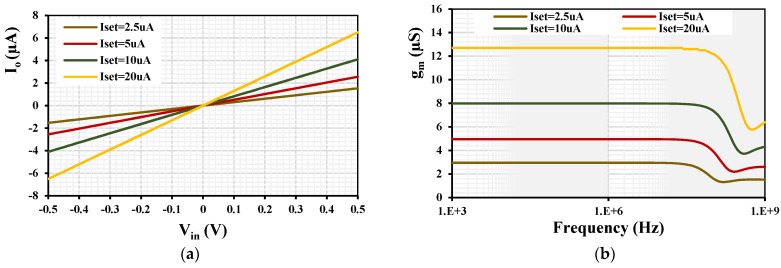
The I_o_ versus V_in_ (**a**) and the transconductance AC characteristic (**b**) with different setting currents.

**Figure 8:**
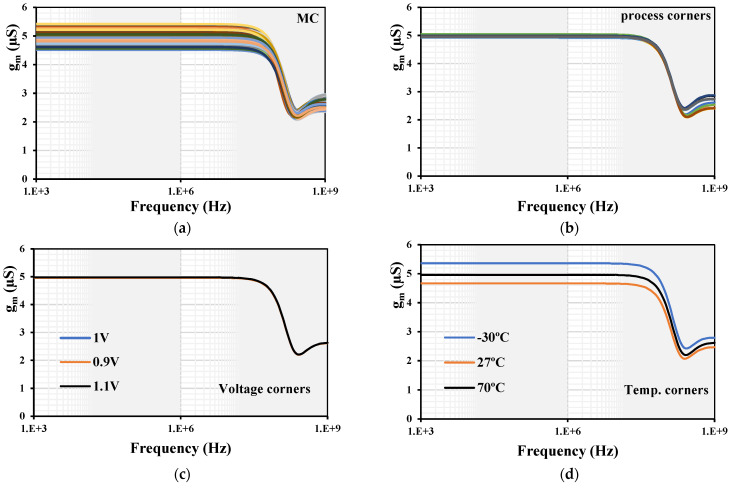
The transconductance AC characteristic of the MI-OTA: (**a**) MC, (**b**) process, (**c**) voltage and (**d**) temperature corners.

To obtain the dynamic characteristic of the MI-OTA, a sine wave of 1 kHz was applied to the input of the OTA. The extended linearity of the MI-OTA with various setting currents I_set_ = (2.5, 5, 10, 20) μA is shown in Figure 7a. The transconductance AC characteristic of the MI-OTA with various setting currents I_set_ = (2.5, 5, 10, 20) μA is shown in Figure 7b. The transconductance was (2.9, 4.9, 7.9, 12.6) μS, respectively. The transconductance AC characteristic with I_set_ = 5 μA was repeated for the Monte Carlo (MC) analysis with 200 runs and process, voltage, and temperature (PVT) corners, as shown in Figure 8.

**Figure 9:**
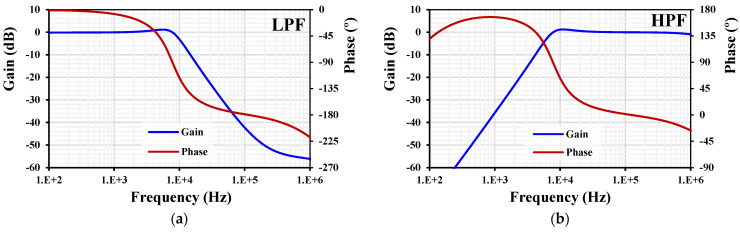

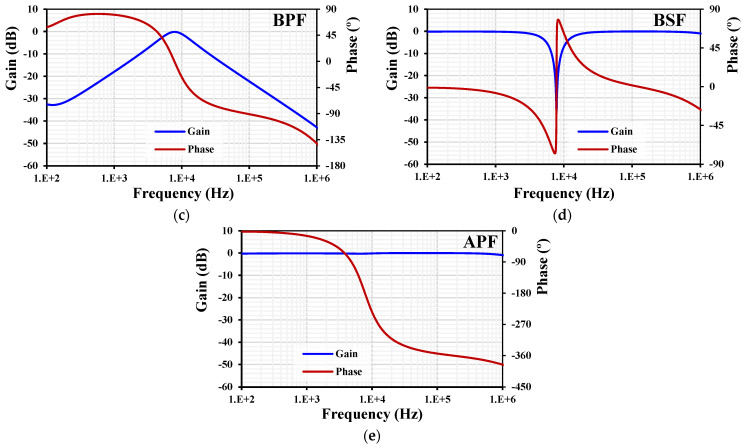
The frequency and phase characteristics of the filter.

**Figure 10:**
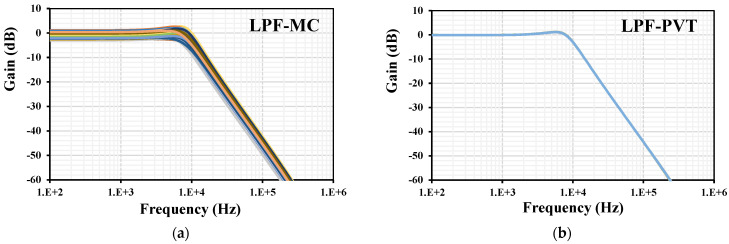
The frequency characteristics of the LP filter with (**a**) MC analysis and (**b**) PVT corners.

The process corners of the transistor were fast–fast, fast–slow, slow–fast, and slow–slow. For the input MIM capacitor C_B_, they were fast–fast and slow–slow. The voltage corners were V_DD_ ± 10%, and the temperature corners were −30 °C and 70 °C. The MC showed min. 4.5 μS and max. 5.4 μS. The process corners showed min. 4.91 μS and max. 5 μS. The temperature corners showed min. 4.66 μS and max. 5.36 μS. The voltage corners showed min. 4.94 μS and max. 4.97 μS. All the transconductance variations were in the acceptable range. The frequency and phase characteristics of the filter with I_set1–4_ = 5 μA and C_1–3_ = 100 pF are shown in Figure 9. The cutoff frequency was 7.85 kHz. The simulation of the LPF was repeated with MC and PVT corners analyses, as shown in Figure 10. While the curves for the PVT overlapped, for the MC, the gain variation at low frequencies was in the range of −3.1 dB to 1.6 dB and the cutoff frequency variation was in the range of 0.72 kHz to 9.3 kHz, which can be realigned by adjusting the setting current.

**Figure 11:**
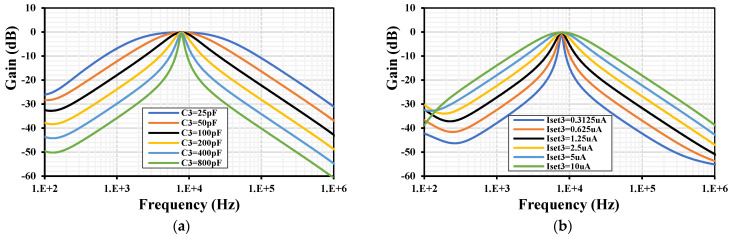
The frequency characteristic of the BPF with different values for (**a**) the capacitor C_3_ and (**b**) the setting current I_set3_.

**Figure 12:**
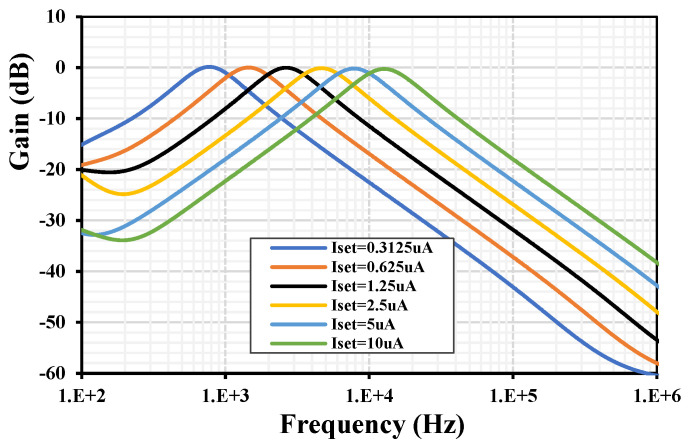
The frequency characteristic of the BPF with different I_set1–4_.

To demonstrate the tuning capability of the *Q*, Figure 11 shows the frequency characteristics of the BPF with: (a) I_set1–4_ = 5 μA, C_1,2_ = 100 pF and tuning C_3_ = (25, 50, 100, 200, 400, 800) pF and (b) with I_set1,2,4_ = 5 μA, C_1–3_ = 100 pF and tuning I_set3_ = (0.3125, 0.625, 1.25, 2.5, 5, 10) μA. To demonstrate the tuning capability of the ω, Figure 12 shows the frequency characteristics of the BPF with C_1–3_ = 100 pF and tuning I_set_ = I_set1–4_ = (0.3125, 0.625, 1.25, 2.5, 5, 10) μA. The natural frequency was (0.767, 1.44, 2.63, 4.62, 7.85, 12.7) kHz, respectively.

**Figure 13:**
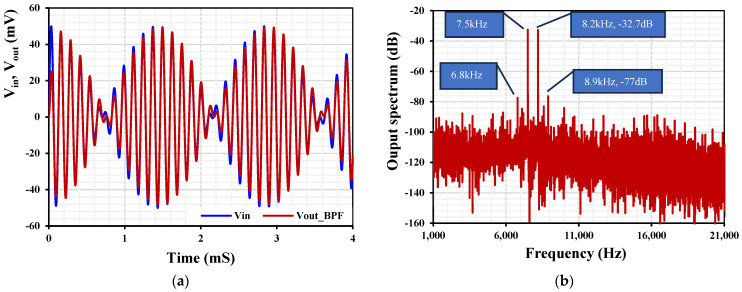
(**a**) The transient characteristic of the BPF and (**b**) the spectrum of the output signal.

To determine the third intermodulation distortion (IMD3) of the BPF, two closed tones were applied to the input of the BPF. Both tones were a sine wave with an amplitude of 25 mV but with different frequencies: 7.5 kHz and 8.2 kHz. The transient analyses of the input and output signal are shown in Figure 13a and the spectrum of the output signal is shown in Figure 13b. The IMD3 was −43.6 dB, which indicates a 0.66% THD.

**Figure 14:**
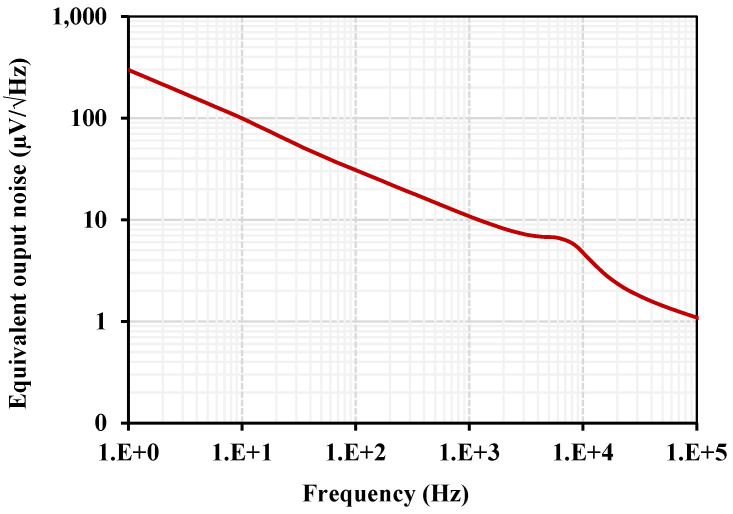
The output noise of the BPF.

The equivalent output noise is shown in Figure 14. The integrated noise in the filter bandwidth of 4.8 kHz to 12.5 kHz was 485.7 μV; hence, the dynamic range DR was calculated to be 40 dB for 1% IMD3.

The OTA-based universal filters in [16,34,41,45,46] were used as a comparison, as shown in Table 3. Compared with the filters in [16,34], which offer independent/electronic control of the ω_o_ and *Q* as well as a high-*Q* filter, the proposed filter offers larger transfer functions that cover both the non-inverting and the inverting transfer functions of the LPF, HPF, BPF, BSF, and APF. Compared with the filters in [41,45,46], which provide sub-volt supply voltage, the proposed filter offers independent/electronic control of the ω_o_ and *Q* and a high-*Q* filter. Compared with the filters in [16,34,41], the proposed filter offers low voltage and low power consumption.

## 4. Conclusions

In this paper, a new multiple-input single-output voltage-mode universal filter using MI-OTAs is proposed. In this filter, the pole-*Q* can be tuned by varying the capacitance and setting current. The natural frequency can also be electronically controlled. The proposed filter uses four MI-OTAs that its differential pair realizes using the multiple-input MOS transistor technique, which does not increase the power consumption of the OTA. This work shows that an MI-OTA-based filter with 10 transfer functions, namely the non-inverting and inverting transfer functions of the LPF, HPF, BPF, BSF, and APF, can be obtained without changing the circuit topology. The proposed filter is suitable for low-voltage-supply, low-power-consumption and low-frequency applications like the biomedical one, since it is capable of operating with a 1 V supply voltage and consumes 120 μW of power for a 5 μA setting current. 

## Figures and Tables

**Table 1:** Obtaining the variant filtering functions of the proposed universal filter.

Filtering Function		Input
LPF	Non-inverting	$V_{in1}$
	Inverting	$V_{in2}$
BPF	Non-inverting	$V_{in3}$
	Inverting	$V_{in4}$
	Non-inverting	$V_{in5}$ $\;\mathrm{or}\;V_{in6}$
	Inverting	$V_{in7}$ $\;\mathrm{or}\;V_{in8}$
HPF	Non-inverting	$V_{in2}=V_{in7}=V_{in9}$
	Inverting	$V_{in1}=V_{in5}=V_{in10}$
BSF	Non-inverting	$V_{in7}=V_{in9}$
	Inverting	$V_{in5}=V_{in10}$
APF	Non-inverting	$V_{in7}=V_{in8}=V_{in9}$
	Inverting	$V_{in5}=V_{in6}=V_{in10}$

**Table 2:** Parameters of the components of the MI-OTA.

Transistor	W/L (μm/μm)
M_1_–M_4_, M_13_–M_18_	10/0.5
M_1SD_, M_2SD_	5/0.5
M_5_–M_12_	20/0.5
M_R_	4/5
C_B_ = 0.5 pF	
V_B1_ = −300 mV, V_B2_ = 200 mV	

**Table 3:** Comparison of the properties of this work with those of high-*Q* universal filters.

Factor	Proposed	[16] 2010	[34] 2019	[41] 2020	[45] 2022	[46] 2022
Number of active devices	4-OTA	3-OTA	5-OTA	5-OTA	8-OTA	3-OTA
Realization	0.18 μm CMOS	BJT (AT&T CBIC-R)	Commercial IC (LT1228)	0.18 μm CMOS	0.18 μm CMOS	0.18 μm CMOS
Number passive devices	3-C	3-C	2-C	2-C	2-C	2-C
Type of filter	MISO	MISO	MIMO	MISO	MIMO	MIMO
Total number of offered responses	12 (VM)	5 (CM)	7 (VM)	20 (MM)	20 (MM)	22 (VM)
Electronic control of $\omega_{o}$	Yes	Yes	Yes	Yes	Yes	Yes
Independent control of *Q*	Yes	Yes	Yes	No	No	No
High-*Q* filter	Yes	Yes	Yes	No	No	No
All-grounded passive devices	Yes	Yes	Yes	Yes	Yes	Yes
High input impedances	Yes	-	Yes	Yes	Yes	Yes
Unnecessary input-matching conditions	Yes	No	Yes	Yes	Yes	Yes
Unnecessary inverted input signal	Yes	No	Yes	Yes	Yes	Yes
Achievable range of *Q*-factor	0.26 to 9.7 ^a^ 0.62 to 9.7 ^b^	1 to 64 ^c^ 1 to 64 ^d^	1.02 to 3.03 ^e^	-	-	-
Power supply (V)	1	±2	±15	±0.9	±0.3	0.5
Power dissipation (nW)	120 × 10^3^	-	861 × 10^6^	177.3 × 10^3^	5770	37
Natural frequency (kHz)	7.85	1000	159.16	3.39 × 10^3^	5	0.153
Total harmonic distortion (%)	1@140mV_pp_	-	-	-	<2@200mV_pp_	0.33@100mV_pp_
IMD3	−43.6 dB	-	−43.6 dBc	-	-	-
Output integrated noise (μVrms)	485.7	-	-	-	115	220
Dynamic range (dB)	40	-	-	-	53.2	50
Verification of result	Sim	Sim	Sim/Exp	Sim	Sim	Sim

Note: ^a^ = the capacitance varies from 25 to 800 pF, ^b^ = the biasing current varies from 0.312 to 10 μA, ^c^ = the capacitance varies from 2 to 128 nF, ^d^ = the biasing current varies from 1 to 70 μA, ^e^ = the g_m_ varies from 1 to 3 mS.

## Data Availability

Data are contained within the article.

## Abbreviations

MI-OTA	multiple-input operational transconductance amplifier
CCII	second-generation current conveyor
CFOA	current feedback operational amplifier
VDIBA	voltage differencing inverting buffered amplifier
SIMO	single-input multiple-output
MISO	multiple-input single-output
MIMO	multiple-input multiple-output
MOS	metal oxide semiconductor
MOST	metal oxide semiconductor transistor
CMOS	complementary metal oxide semiconductor
GD	gate-driven
MIBD	multiple-input bulk-driven
TSMC	Taiwan Semiconductor Manufacturing Company
VM	voltage-mode
CM	current-mode
MM	mixed-mode
LPF	low-pass filter
HPF	high-pass filter
BPF	band-pass filter
BSF	band-stop filter
APF	all-pass filter
